# Supplementary material for: Don’t dismiss logistic regression: the case for sensible extraction of interactions in the era of machine learning
Source: BMC Med Res Methodol. 2020 Jun 29;20:171. doi: 10.1186/s12874-020-01046-3 (PMC7325087; doi:10.1186/s12874-020-01046-3)
Supplement: Supplementary file 1 — Additional file 1 Appendix Table 1. Glossary of Terms. Appendix Table 2. Pros and Cons of Logistic Regression, Decision Tree, and Random Forest Approaches. Appendix Figure 2. Characteristics of linear modeling approach (blue), decision trees (orange), random forest (green), original generated points (black); (a-b) Predictor X versus response Y for the a) linear continuous predictor, b) non-linear transformed predictor. Appendix Figure 2. Characteristics of generalized linear modeling approach (green), decision trees (purple), random forest (red); (a-b) Predictors X1 and X2 versus binary response Y for the a) linear continuous predictors, b) non-linear transformed predictors. Appendix Figure 3. Decision Diagram for Application of Hybrid Approach. Appendix Figure 4. Performance of Hybrid Approach for select datasets is on par with Random Forest for domain where Random Forest outperforms Logistic Regression by at least 2%; (a) 37 datasets selected with highest positive residual from model regressed on performance gains for Hybrid and Random Forest approaches in the aforementioned domain; (b) Boxenplots of predictive performance of the Logistic Regression, Random Forest and Hybrid methods for these 37 datasets; other comparison methods enforce stricter thresholds/cutoffs for performance gain. Appendix Figure 5. Comparison of Additional Machine Learning Modeling Approaches Across: (a) 277 benchmark datasets featured in the main text; (b) the 77 datasets that remain after selection of datasets where Random Forest significantly outperforms the traditional modeling approaches; the threshold for outperformance for Random Forest was greater than that utilized in previous comparison approaches for effect. Appendix Table 3. Comparison of median AUROC for each machine learning and statistical modeling approach across 277 benchmark datasets and a subset of these datasets where Random Forest significantly outperforms the traditional modeling approaches (n = 77); 95% confidence int [file 12874_2020_1046_MOESM1_ESM.docx]

**Appendix**

**Appendix Table 1: Glossary of Terms**

| Statistical Term | Machine Learning Term | Definition |
| --- | --- | --- |
| Predictor(s)/Covariate(s) | Feature; Feature Vector | Independent variable used to predict outcome variable. |
| Observation/Sample | Instance/Sample | Actualization/value/instances of a particular variable of interest. |
| C-statistic, Concordance statistic, AUROC | AUROC | Concordance measure for the overall goodness of fit or predictive performance of selected model over the full range of prediction thresholds for a binary outcome. |
| Parameters | Weights/Tuning constants | In statistics, a parameter is a numerical value that serves to characterize a population. In machine learning, the parameter or weight is an argument of a function or model that is optimized given the set of data to increase predictive performance on unseen data. Both are estimated using training data. |
| Risk-ratio |  | The ratio of probabilities of an event at two different values of the predictors |
| Odds-ratio |  | The ratio of the odds of an event at two different values of the predictors |
| Interaction-effect |  | The change in the effect of one predictor on the outcome across the value(s) of another predictor(s) |
| Response | Label | Outcome or dependent variable to predict from the predictors. |
| Estimation/Fitted model | Learning | The process of learning the parameters of the specified model. |
| Likelihood function | Fitness/Cost/Objective/Loss | The objective function defined by the criteria on which the model parameters are estimated or learned |
| Classification/Regression | Supervised learning | Learning the model parameters given the predictors and response variables |
| Clustering/Dimensionality Reduction/Density Estimation | Unsupervised learning | Learning model parameters in the absence of outcome observations or a gold standard. |
| Test set performance | Generalization to unseen data | Evaluation of the model on unseen data that was not used for fitting the model parameters or hyperparameters to estimate how well the model recovers the real-world. Typically, model interpretability techniques would be applied on this set of data using a machine learning model, while in traditional statistics, the model parameters estimated using the training data are directly interrogated. |
| Overfitting | Overfitting/Memorization/High Variance | The condition from which a highly complex model is too closely fit to a collection of data points such that the model performs poorly on unseen data. |
| Lack-of-fit/Omitted variables | Underfitting/High Bias | When the specified model is missing terms that ought to be included (i.e., are in the data generating process). |
| Logit or sigmoid link function | Sigmoid transform/activation function | One type of variable transformation that seeks to transform a continuous predictor or set of predictors into a probability for binary outcomes. Activation functions apply an additional non-linear functional form to an input. |
| Residuals/Residual sum of squares/Deviance statistic | Loss/Cost | A measure of divergence between the observed and predicted outcomes that indicates how well the statistical model or specified algorithm fit the given data. |
| Penalization term such as in ridge regression (L2) or Lasso (L1) penalization  Bayesian prior variance or scale parameter | Hyperparameters such as number of CART estimators, depth of tree, size of hidden layers | A set of parameters that pertain to the procedure used to estimate the model that are not part of the model itself. For example, tuning parameters or hyperparameters. These are often selected using cross-validation in machine learning. In machine learning, evaluation of the hyperparameters on the validation set that is not used to estimate the model parameters is used to evaluate how well the model specification may generalize to unseen data. In statistics, typical hyperparameters may include the prior-parameters that define the prior distribution. In machine learning, the tuning parameters may govern how much to regularize a particular model. |
| Propensity weighting | Class Balancing | A procedure in machine learning where the model is trained by either: oversampling instances of under-expressed classes, under-sampling over-expressed classes, or adding a weighting term that penalizes the model more for making incorrect predictions on the minority classes. |
| Model fit | Embedding/Projection | Low dimensional subspace from which the data can be represented in such a way that maintains fidelity to the original source. |
| Random Noise | Data Augmentation/ Regularization/Penalization | Techniques to modify the input data such as adding noise to the predictors and outcome labels, random variable transformations, sampling from a latent distribution or imposing a prior over the parameters that seek to make the model more generalizable to unseen data. |
| Binomial/Multinomial | Binary/Multiclass | A description for the number of outcome categories for a classification problem. |
| Logistic Regression | Single Layer Perceptron with Sigmoid/Softmax Activation | Description of equivalent modeling approach in statistics/machine learning, where a dichotomous/nominal dependent variable is predicted from a set of predictors; a logit link function is applied to a linear combination between the predictor and parameters to form the final probability |

* We note here that the aforementioned terms are analogous yet not entirely identical between the disciplines of machine learning and statistics.

**Appendix Table 2:** Pros and Cons of Logistic Regression, Decision Tree, and Random Forest Approaches

| Logistic Regression | |
| --- | --- |
| Pros | **Cons** |
| Simple, intuitively explained model | Difficulties in handling non-linearity |
| Best captures linearly separable data | Selection of interactions difficult to specify |
| Not prone to overfitting; minimal hyperparameter tuning necessary | Failure to make inferences under highly collinear conditions; selection of features necessary |
| Decision Trees | |
| Pros | **Cons** |
| Decision making is transparent for small number of predictors and has low bias | High variance in estimates |
| Random Forest | |
| Pros | **Cons** |
| Handles non-linearities and interactions in data; captures complex patient heterogeneity/comorbidities | Prone to over-fitting/sensitive to the properties of the dataset |
| Handles missing data well | Difficult to interpret / understand which predictors are important |
| Unaffected by scaling of predictors | Over-specification of hyperparameters |
| Decorrelates decision trees to arrive at low variance estimate | Requires heavy computational resources |

**Contrasting Linear Modeling and Machine Learning for Continuous Outcomes**

In Appendix figure 1a, we have a use case where we have a single linear continuous predictor. The distribution of the response is generated conditionally on this input by the true model:

$$Y=9X$$

$$X\sim U(-3,3)$$

It seems upon inspection that a line passing through the datapoints could best describe this relationship, so first we fit a linear regression model to the data. The derived slope was found to be exactly 9 and the model had a mean absolute residual (MAR) of 0 on a held-out validation set. This example depicts the case when the true model is a linear regression model. Now, we fit two CART-models, a decision tree and the random forest model, and we see that the true linear relationship was approximated by these machine learning approaches using a staircase type fit. Under this model, the machine learning approach is guaranteed to have a higher residual error, and upon inspection of held out test data, this is the case (MAR of decision tree and random forest of 0.38 and 0.29 respectively). Now, in Appendix figure 1b, we transform the continuous predictor X into the dependent outcome variable Y via the model:

$$Y=-2*\mathbb{1}_{X<-\mathbb{2}}+sin\left( X \right)-\mathbb{1}_{\left| X \right|\mathbb{<1}}$$

$$X\sim U(-3,3)$$

We see that the linear fit cuts through the middle of the entire dataset and as such has a worse goodness of fit (mean absolute residual of 0.46) than the machine learning approaches (0.07 and 0.05 for decision tree and random forest respectively).

**Appendix Figure 2:** Characteristics of linear modeling approach (blue), decision trees (orange), random forest (green), original generated points (black); (a-b) Predictor X versus response Y for the a) linear continuous predictor, b) non-linear transformed predictor;

**Contrasting Logistic Regression and CART Models with More than One Predictor**

In appendix figure 2a, we have a use case where we have two linear continuous predictors and a binary endpoint. The distribution of the response is defined implicitly by the following data generating process:

$$Y=\mathbb{1}_{X_{\mathbb{2}}>X_{\mathbb{1}}}$$

$$X_{1}\sim U\left( -3,3 \right)$$

$$X_{2}\sim U\left( -3,3 \right)$$

Clearly, the identity line, $X_{1}=X_{2},$ represents the true decision boundary between predicting a positive outcome (Y=1; when $X_{2}$is greater than $X_{1}$) and a negative outcome (Y=0; when $X_{2}$is less than $X_{1}$). As such, we fit a logistic regression model to the data with the aim that it would capture the true decision boundary and found that the logistic regression model was able to accurately capture the binary end points with 100% accuracy on a held-out validation set. This example depicts the case when the true model is a logistic regression model. To the same data, we fit two CART-models, a decision tree and the random forest model, and find that the true linear decision boundary between the positive and negative classes was approximated by these machine learning approaches using a staircase type fit. Under this model, the two machine learning models demonstrated lower binary classification accuracy than the linear approach (accuracy of the decision tree was 97.2% and of the random forest was 97.9%).

In another example, appendix figure 1b, we transform the continuous predictors $X_{1}$ and $X_{2}$ into the binary dependent outcome variable Y via the model:

$$Y=\mathbb{1}_{0.5*sin\left( 4X_{1} \right)-0.25*X_{2}+0.25*X_{1}>0}$$

$$X_{1}\sim U\left( -3,3 \right)$$

$$X_{2}\sim U\left( -3,3 \right)$$

Assuming we do not transform the first predictor via the parameterized sine wave introduced in the above simulation, we see that the logistic regression fit cuts through the middle of the entire dataset and as such has a worse goodness of fit (binary classification accuracy of 80.1%) than the machine learning approaches (96.8% and 98% for decision tree and random forest respectively).

**Appendix Figure 2:** Characteristics of generalized linear modeling approach (green), decision trees (purple), random forest (red); (a-b) Predictors $X_{1}$ and $X_{2}$ versus binary response Y for the a) linear continuous predictors, b) non-linear transformed predictors

**Decision to Apply Hybrid Approach**

**Appendix Figure 3: Decision Diagram for Application of Hybrid Approach**

**Appendix Figure 4: Performance of Hybrid Approach for select datasets is on par with Random Forest for domain where Random Forest outperforms Logistic Regression by at least 2%; (a)** 37 datasets selected with highest positive residual from model regressed on performance gains for Hybrid and Random Forest approaches in the aforementioned domain; **(b)** Boxenplots of predictive performance of the Logistic Regression, Random Forest and Hybrid methods for these 37 datasets; other comparison methods enforce stricter thresholds/cutoffs for performance gain

**Description of Kernel-Based Approaches**

A kernel is a measure of similarity between two observations, $\vec{X_{i}}$ and $\vec{X_{j}}$ , as defined by the functional mapping $k: \chi\times\chi\mathbb{\to R}$, or $k\left( \vec{X_{i}},\vec{X_{j}} \right)=\left\langle\vec{X_{i}} | \vec{X_{j}} \right\rangle_{\mathcal{V}}$, an inner product in another space $\mathcal{V}$. Popular examples include the linear ($k\left( \vec{X_{i}},\vec{X_{j}} \right)$=$\vec{X_{i}}\cdot\vec{X_{j}}$) and cosine ($k\left( \vec{X_{i}},\vec{X_{j}} \right)$=$\frac{\vec{X_{i}}\cdot\vec{X_{j}}}{\left\| \vec{X_{i}} \right\|\left\| \vec{X_{j}} \right\|}$) kernels. Kernel-based approaches posit that a statistical or machine learning problem that may not have a linearly separable solution in a lower dimensional vector space may be linearly separable in a higher dimensional space. Thus, these methods seek to transform the set of low-rank predictors $\vec{X}$ into a higher dimensional predictor space via the mapping $\phi: \chi\to\mathcal{V}$. However, the direct computation of the higher dimensional set of predictors may be infeasible. A “kernel trick”, which calculates the inner product $k\left( \vec{X_{i}},\vec{X_{j}} \right)=\left\langle\phi\left( \vec{X_{i}} \right) | \phi\left( \vec{X_{j}} \right) \right\rangle_{\mathcal{V}}$, circumvents the explicit calculation of the higher dimensional vector and transforms the original low-rank design matrix, $X$ into a high-rank matrix $K$, of which the rows represent the observations and the columns/predictors are the pairwise kernel between one observation and each of the set of observations. The new predictor space is thus comprised of comparisons between the observations to represent the linearly separable high dimensional space. Here, we describe a few kernel-based approaches that we included in comparisons to the hybrid approach.

**Support Vector Machines**

Support vector machines (SVM) attempt to establish a hyperplane that can best partition this space by the correct outcome classes for binary or multinomial outcome variables. This is actualized through definition of the hyperplane that has maximal separation, or the largest margin, from the closest observations (termed support vectors) of different classes:

$$\vec{w}\cdot\phi\left( \vec{X} \right)+b=0$$

Where $\vec{w}$ is normal to the hyperplane and the distance to this hyperplane from the support vectors $\vec{X_{0}}$ is given by:

$$d\left( \vec{X_{0}} \right)=\frac{\left| \vec{w}\cdot\phi\left( \vec{X_{0}} \right)+b \right|}{\left\| \vec{w} \right\|}$$

The optimal hyperplane is able to minimize this distance while correctly classifying these observations, which is parameterized and solved by the following objective function ($y_{n}$ is equal to 1 for positive instances and -1 for negative instances, $\xi_{i}$ penalizes the model for incorrect terms):

$$w^{*}=argmax_{W,b,\xi}\frac{1}{\left\| W \right\|_{2}}+C\sum_{i} \xi_{i}$$

$$s.t. \left[ min_{n}y_{n}\left( \vec{w}\cdot\phi\left( \vec{X_{n}} \right)+b \right) \right]$$

Since $\phi\left( \vec{X_{n}} \right)$ may be impossible to specify, this expression can be reformulated in terms of the kernel function to present a tractable solution. As a method for comparison to our hybrid method, we utilized a support vector machine classifier with a Radial Basis Function (RBF) kernel, given by:

$$k\left( \vec{X_{i}},\vec{X_{j}} \right)=exp\left( -\gamma\left\| \vec{X_{i}}-\vec{X_{j}} \right\|^{2} \right)$$

**Kernel Logistic Regression**

Kernel Logistic Regression (KLR) is configured similarly to its traditional statistical counterpart but utilizes the kernel matrix $K$ instead of its default predictor design matrix $X$. Thus, the coefficients of this kernel-approach are not interpretable. To predict on new data, the kernel similarity is computed pairwise between each testing observation and every training observation. Since the resulting matrix is high-rank, kernel approximation approaches such as the Nystrom method can effectively subsample the kernel matrix to arrive at a lower-rank approximation that can be employed in a scalable manner. We utilized the ANOVA kernel, which in functional form is similar to the RBF kernel and applied the Nystrom method to generate a low-rank approximation of this kernel for our implementation of Kernel Logistic Regression for comparison to the hybrid approach.

**Deep Learning**

While deep learning is not explicitly a kernel-based method, these approaches, through the use of Artificial Neural Networks (ANN), map high-dimensional representations of the data, of which the predictors are represented by nodes or neurons (not to be confused with their biological counterpart), to successive layers of nodes that are able to arrive at more linearly separable solutions, and finally to an output layer that presents probabilities for obtaining each class. The latent representation of an intermediate layer is $\vec{Z}$, which is represented by the following mathematical notation:

$$\vec{Z}=\sigma\left( W\vec{X}+\vec{b} \right)$$

Where $\sigma$ is a link function such as the Logit/Sigmoid transform. This is of similar functional form to the multinomial logistic regression model; however, the transformed data is then further transformed through subsequent applications of many of these types of layers to arrive at a final prediction, and all parameters of the model are optimized through gradient descent to reduce the divergence from the observed and expected outcomes, as measured through objective functions such as the Mean Squared Error ($MSE=\left\| y_{c}-\hat{y_{c}} \right\|_{2}$) or Cross Entropy ($CE=-\sum_{c\in classes} y_{c}log\left( P\left( y_{c} | \vec{Z} \right) \right)$) losses.

**Description of LASSO-Based Interaction Model**

Here, we describe how LASSO can be applied to select interactions to be used in a final logistic regression model, as a means to compare the performance and transparency of interaction selection approaches. When the number of predictors far outnumbers the number of observations (such as when modeling all pairwise interactions), the data becomes inherently multi-collinear, meaning that some of the predictors can be predicted from other predictors and are thus not independent. This reduces the inferential capacity of the model. As such, LASSO applies L1-penalization to the logistic regression model (adding the L1-norm of the model coefficients to the objective function), which essentially serves to eliminate collinearity terms by reducing their coefficients to 0:

$${min_{\beta} \lambda\left\| \beta\right\|}_{1}+ \frac{1}{N} \sum_{i} log\left( exp\left( y_{i}-\vec{\beta}^{T}\vec{X_{i}} \right)+1 \right)$$

We extracted all pairwise interactions for the predictors of every dataset for the 277 datasets of the comparison study and subsequently fit an L1-penalized logistic regression model to each dataset and recorded predictive performance. We note that the coefficients of the LASSO derived model are unable to be utilized for statistical inference, and as such presents limited interpretability. For our first case study in the main text, we resolved this issue by retaining the non-zero terms after application of LASSO and then fit a normal logistic regression model to the final predictor set.

**Comparison of Kernel and LASSO-Based Approaches to Hybrid Approach**

Here, we discuss the results for training and cross-validating naïve implementations of the aforementioned techniques (SVM, KLR, ANN, LASSO) on the 277 datasets of this study and compare results to traditional Logistic Regression, Random Forest and our Hybrid Model. We expect our hybrid approach to outperform the Logistic Regression approach in cases from which the performance of Random Forest is much greater than the generalized linear modeling approach while achieving performance on-par with the kernel and LASSO approaches, which are complex model specifications designed to achieve high predictive performance at the expense of interpretability. We utilized Mann-Whitney U tests with Bonferroni multiple hypothesis correction to compare the median performance between the modeling approaches across the datasets. Here, we abbreviate Logistic Regression as LR and Random Forest as RF.

**Appendix Figure 5: Comparison of Additional Machine Learning Modeling Approaches Across: (a)** 277 benchmark datasets featured in the main text; **(b)** the 77 datasets that remain after selection of datasets where Random Forest significantly outperforms the traditional modeling approaches; the threshold for outperformance for Random Forest was greater than that utilized in previous comparison approaches for effect

Across the 277 datasets featured in the main text, we found comparable performance between all approaches save for the Random Forest Modeling approach. We suspect that the superior performance of the random forest model can be attributed to the emphasis placed on modeling interactions over non-linearity, as would be more emphasized by the kernel approaches. When we subset the datasets by the domain from which the performance of the random forest model exceeded that of the logistic regression (set to RF AUROC > 0.8, RF AUROC – LR AUROC > 0.1), the LASSO and LR approaches experienced notable reductions in their AUROC and were unable to match the performance of the top performing machine learning approaches outside of Random Forest. Meanwhile, the Hybrid Approach was still able to achieve performance on par with the other top performing kernel methods, as implied through non-parametric statistical testing.

**Appendix Table 3:** Comparison of median AUROC for each machine learning and statistical modeling approach across 277 benchmark datasets and a subset of these datasets where Random Forest significantly outperforms the traditional modeling approaches (n=77); 95% confidence intervals were derived using a 1000-sample non-parametric bootstrap; the threshold for outperformance for Random Forest was greater than that utilized in previous comparison approaches for effect

| **Modeling Approach** | **277 Dataset Study (n=277); Median AUROC** | **Domain where RF Surpasses LR (n=77); Median AUROC** |
| --- | --- | --- |
| **KLR** | 0.79±0.028 | 0.77±0.022 |
| **LASSO** | 0.79±0.026 | 0.73±0.038 |
| **ANN** | 0.74±0.028 | 0.72±0.026 |
| **SVM** | 0.78±0.032 | 0.77±0.042 |
| **LR** | 0.77±0.032 | 0.71±0.0092 |
| **Hybrid** | 0.78±0.022 | 0.75±0.0148 |
| **RF** | 0.9±0.0164 | 0.93±0.0132 |

**Appendix Table 4:** Pairwise comparison of median AUROC for each of the modeling approaches for the 277 benchmark datasets; 2-tailed non-parametric Mann-Whitney U tests were corrected using family-wise error rate Bonferroni correction to find median AUROCs that were different between the modeling approaches on the 0.05 alpha significance level; we note here that there still exists 61 datasets from which the Hybrid approach is still able to outperform the Random Forest Approach

| **Method A** | **Method B** | **P-Value** | **Reject** |
| --- | --- | --- | --- |
| KLR | LASSO | 1.000 | FALSE |
| KLR | ANN | 0.058 | FALSE |
| KLR | SVM | 1.000 | FALSE |
| KLR | LR | 1.000 | FALSE |
| KLR | Hybrid | 1.000 | FALSE |
| KLR | RF | 0.000 | TRUE |
| LASSO | ANN | 0.808 | FALSE |
| LASSO | SVM | 1.000 | FALSE |
| LASSO | LR | 1.000 | FALSE |
| LASSO | Hybrid | 1.000 | FALSE |
| LASSO | RF | 0.000 | TRUE |
| ANN | SVM | 0.461 | FALSE |
| ANN | LR | 0.969 | FALSE |
| ANN | Hybrid | 0.075 | FALSE |
| ANN | RF | 0.000 | TRUE |
| SVM | LR | 1.000 | FALSE |
| SVM | Hybrid | 1.000 | FALSE |
| SVM | RF | 0.000 | TRUE |
| LR | Hybrid | 1.000 | FALSE |
| LR | RF | 0.000 | TRUE |
| Hybrid | RF | 0.000 | TRUE |

**Appendix Table 5:** Pairwise comparison of median AUROC for each of the modeling approaches for a subset 277 benchmark datasets from which the Random Forest outperforms the traditional modeling approaches (n=77); 2-tailed non-parametric Mann-Whitney U tests were corrected using family-wise error rate Bonferroni correction to find median AUROCs that were different between the modeling approaches on the 0.05 alpha significance level

| **Method A** | **Method B** | **P-Value** | **Reject** |
| --- | --- | --- | --- |
| KLR | LASSO | 0.012 | TRUE |
| KLR | ANN | 0.058 | FALSE |
| KLR | SVM | 1.000 | FALSE |
| KLR | LR | 0.000 | TRUE |
| KLR | Hybrid | 0.121 | FALSE |
| LASSO | ANN | 1.000 | FALSE |
| LASSO | SVM | 0.017 | TRUE |
| LASSO | LR | 1.000 | FALSE |
| LASSO | Hybrid | 1.000 | FALSE |
| ANN | SVM | 0.121 | FALSE |
| ANN | LR | 1.000 | FALSE |
| ANN | Hybrid | 1.000 | FALSE |
| SVM | LR | 0.000 | TRUE |
| SVM | Hybrid | 0.712 | FALSE |
| LR | Hybrid | 0.000 | TRUE |

**Inferential Capacity of LASSO Selection of interactions from Case Study 1**

While the aforementioned approach demonstrated that sensible extraction of interactions can achieve performance on par of other competing machine learning approaches, we also demonstrate the inferential advantages of our Hybrid Approach over that of other interaction selection methods. We extend the LASSO modeling approach by utilizing it to select main effects and interactions from the epistasis dataset featured from the second case study. The new set of predictors identified by the LASSO model were used to train a final logistic regression model for statistical inference. Performance of the logistic regression model based on LASSO-selected predictors (AUROC 0.771±0.0474) was on par with the random forest approach; the LASSO-based approach performed noticeably worse than that of the hybrid approach. Furthermore, while the top two predictors identified by the LASSO-selected logistic regression model were correspondent to that obtained from the hybrid approach, the selection model failed to incorporate the main effects and also failed to include other previously identified important interactions.

**Appendix Table 6:** Top 10 largest odds-ratios of the Epistasis Logistic Regression Model coefficients after using LASSO to select predictors from a logistic regression model which included all pairwise predictors; note how all main effects have been excluded from this list

| **Predictors** | **Odds-Ratio** |
| --- | --- |
| **P1_1:P2_1** | 30.739549 |
| **P1_1:P2_0** | 4.186583 |
| **N11_0:N14_1** | 1.788621 |
| **N7_1:N13_1** | 1.687043 |
| **N2_0:N5_0** | 1.669593 |
| **N1_1:N12_0** | 1.609355 |
| **N4_1:N16_2** | 1.603160 |
| **N0:N8_1** | 1.529902 |
| **N3_0:N6_0** | 1.491766 |
| **N2_1:P1_2** | 1.490631 |

**Meta-Learning for Decision to Apply Hybrid Approach**

As mentioned in the main text, our Hybrid Approach confers additional performance gains for linear modeling approaches when the machine learning / Random Forest model is able to outperform its traditional counterpart. While subtracting the goodness-of-fit statistics between both models can serve to help inform the user of when to utilize these techniques, there may be specific aspects of the dataset that can be telling when it comes to making this decision. As such, we sought to study which characteristics of the dataset (eg. entropy of class labels, noise to signal ratio, dependency structure) could predict when the random forest model would outperform the linear approach and that being would warrant the inclusion of the hybrid modeling approach. Using the OpenML API, we extracted key descriptive features (some of which were aforementioned; descriptions of these features can be found here: <https://www.openml.org/search?type=measure> ) for each of the 277 datasets featured in the study, imputed missing statistics using Multivariate Imputation by Chained Equations (MICE), and utilized this information to train a random forest classifier to predict when the AUROC of the random forest model would exceed that of the logistic regression model by at least 0.1. Using leave-one-out cross validation, we obtained an AUROC of 0.90 and F1-Score of 0.84 for the prediction of when the random forest model would outperform the logistic regression. An inspection of the cluster plot of meta-predictors derived from the 277 datasets demonstrates meaningful clustering of similar meta-features and datasets that yielded this performance gain from the machine learning approach. Using the Gini Coefficient derived from the mean purity in labels that resulted from the binary splits for each meta-feature, we were also able to assess which meta-features were important in suggesting the use of the hybrid approach. For instance, the equivalent number of attributes (minimum number of predictors necessary to solve modeling task) was found to be important and is a derived measure of how many predictors should be utilized to describe the binary outcome. As another example, the kurtosis (amount of probability in the tails; a sign of outliers) of the dataset appeared to be predictive of whether the random forest model would outperform the linear approach.

All of this suggests that the characteristics of the dataset alone may be useful enough to decide whether a hybrid approach is warranted, though additional validation is necessary to affirm this notion. Such a meta-estimator can potentially be trained on the performance statistics of previous studies that are related to the user’s study of interest and can then be used to suggest modeling approaches that explore tradeoffs between interpretability and performance.


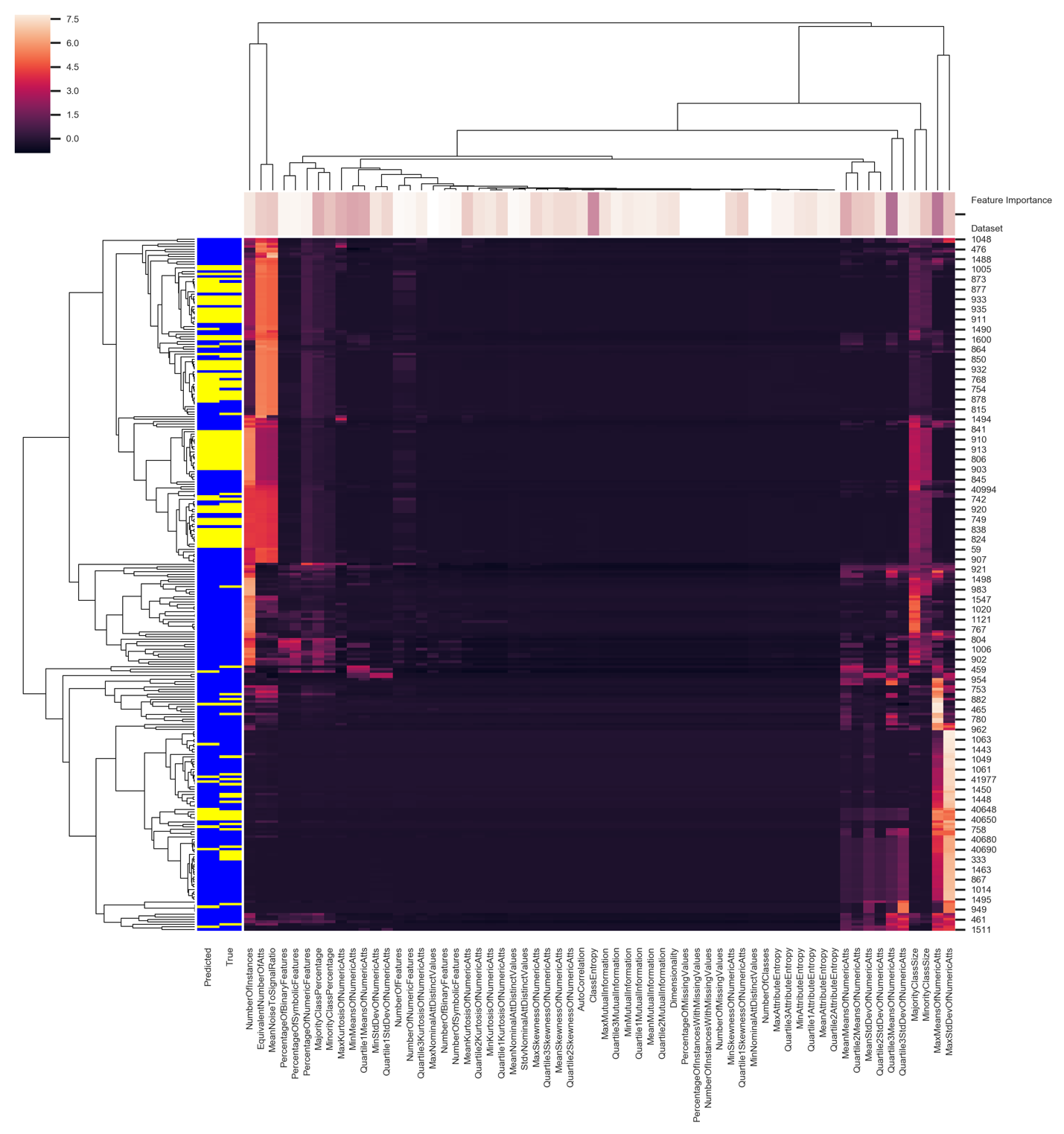


**Appendix Figure 6:** Cluster Heatmap of dataset characteristics of the 277 datasets; rows of matrix indicate datasets; columns of matrix indicate different properties of the dataset; columns were standardized to illustrate trends; left-hand yellow and blue labels were applied to indicate whether Random Forrest model outperformed Logistic Regression model (yellow for gain in performance; blue for no gain in performance, prediction to left of true difference in performance) for a given dataset; column colors indicate relative importance of dataset characteristics as identified by the Gini index

**Discussion of the Rare Events Issue**

The rare events issue concerns itself with the impact of the imbalance of outcome classes on the estimation of the probabilities of registering an event. In the case where one class has a disproportionate number of samples (majority class) versus the class with rare events (minority class), the approximations inherent in the strategy of plugging the estimated parameter into the logistic regression probability functional may lead to predictions and thus classifications that are non-trivially biased towards the majority class. Many machine learning approaches attempt to circumvent the case of rare outcomes by over-sampling observations from the minority class, under-sampling observations from the majority class, generating new observations with the statistical properties of the minority class, or reweighing the objective function of the modeling approach. ​

In the biomedical setting, the rare events issue is much better overcome in the design of a study and should consider whether the study target is to either make predictions or make inferences. With a fixed budget, the most efficient way to spend it is to try and get a balanced sample of cases and controls. Then, one could adjust for the sampling design during the analysis stage. If there is too much data for the estimation algorithm to handle, then we recommend sub-sampling on the cases, and adjusting based on the sampling design. If given the choice between acquiring more controls versus not getting more data, which could potentially further skew the distribution of outcomes, one should opt to include more data. However, the researcher must think carefully about how to compute predictions and on the distinction between the coefficients of the model as a target of inference versus making a prediction. As aforementioned, many of the algorithms attempt to adjust for unbalanced data by reweighing the objective function, which may miss-calibrate the objective for the problem and reduce its inferential and predictive capacity.

In the absence of these unadjusted resampling methods, ​or statistical predictions that average over the uncertainty in the estimated model parameters (e.g., Bayesian posterior means of the logistic regression probability expression), predicted probabilities are ​approximated by plugging in the estimated coefficients into the logistic regression model at prediction time. An ideal threshold for determining whether the predicted probability is sufficient to classify the sample as an event may be determined by a sensitivity analysis of the receiver operating curve (ROC).

However, the aforementioned analyses ignore the uncertainty in the coefficients. Prior literature has discussed circumventing the issues of bias in the coefficients and the failure to incorporate this uncertainty in statistical inference through Bayesian computation, which averages over the uncertainty in the model parameters when computing the optimal estimator for any given loss function.
